# Intrafollicular fibroblast growth factor 13 in polycystic ovary syndrome: relationship with androgen levels and oocyte developmental competence

**DOI:** 10.1186/s13048-018-0455-3

**Published:** 2018-09-26

**Authors:** Yu Liu, Shengxian Li, Tao Tao, Xiaoxue Li, Qinling Zhu, Yu Liao, Jing Ma, Yun Sun, Wei Liu

**Affiliations:** 1 Department of Endocrinology, South Campus, Renji Hospital, School of Medicine, Shanghai Jiaotong University, Shanghai, 201112 China; 2 Department of Endocrinology, Renji Hospital, School of Medicine, Shanghai Jiaotong University, Shanghai, 200127 China; 3 Center for Reproductive Medicine, Renji Hospital, School of Medicine, Shanghai JiaoTong University, Shanghai, 200135 China; 4 Shanghai Key Laboratory for Assisted Reproduction and Reproductive Genetics, Shanghai, 200135 China

**Keywords:** Fibroblast growth factor 13, Testosterone, Follicular fluid, Polycystic ovary syndrome

## Abstract

**Background:**

Fibroblast growth factor 13 (FGF13) is one of the most highly expressed FGF family members in adult mouse ovary. However, its precise roles in ovarian function remain largely unknown. We sought to evaluate the associations between FGF13 in follicular fluid and oocyte developmental competence in patients with polycystic ovary syndrome (PCOS).

**Methods:**

A cross-sectional study was conducted on 43 patients with PCOS and 32 non-PCOS patients who underwent in vitro fertilization/intracytoplasmic sperm injection treatments. The highest quartiles of follicular fluid (FF)-FGF13 (≥117.51 pg/mL) and FF-total testosterone (FF-TT) (≥51.90 nmol/L) were defined as “elevated” FF-FGF13 levels and “elevated” FF-TT levels, respectively.

**Results:**

The levels of FF-FGF13 were skewed, with a median of 82.97 pg/mL (59.79–117.51 pg/mL) in 75 patients. The prevalence of elevated FF-TT levels was significantly higher in the PCOS patients with elevated FF-FGF13 levels than in those without (64.3% vs. 35.7%, adjusted *P* = 0.0096). FF-TT and increased ovarian volume (> 10 mL for one or both ovaries) were positively correlated with FF-FGF13 in PCOS patients (*r* = 0.37, *P* = 0.013; *r* = 0.33, *P* = 0.032). A negative association was evident between FF-FGF13 and the MII oocyte rate in the multiple linear regression analysis (β = − 0.10, SE = 0.045, adjusted *P* = 0.027). However, the associations were not evident in the non-PCOS patients.

**Conclusions:**

Our study suggests the presence of intrafollicular FGF13 in PCOS patients and implies that FGF13 might be involved in the pathophysiological process of PCOS.

**Electronic supplementary material:**

The online version of this article (10.1186/s13048-018-0455-3) contains supplementary material, which is available to authorized users.

## Background

The control of ovarian function is highly complex and often involves multiple endocrine and paracrine signaling factors. In addition to pituitary gonadotrophins, several families of growth factors, such as insulin-like growth factors and transforming growth factors, play crucial roles in ovarian function [1–4].

The fibroblast growth factor (FGF) family, comprising 18 secreted proteins and four intracellular proteins (FGF11–14), participate in the regulation of ovarian function and follicular development [5, 6]. For example, FGF2 promotes granulosa cell proliferation and affects ovarian steroidogenesis [7, 8]. Furthermore, FGF18 is involved in the apoptosis of ovarian granulosa cells [9, 10].

The expression of FGF13 in the murine ovary was reported as early as 1997 [11]. In addition to FGF1 and FGF12, FGF13 is a member of the FGF family that is highly expressed in the adult mouse ovary [12]. FGF13 mRNA is detectable in the corpora lutea, theca and granulosa cells of bovine antral follicles. Moreover, FGF13 mRNA expression is upregulated in the theca cells of the bovine ovary during antral follicle development [13]. Nevertheless, the precise roles of FGF13 in ovarian physiology remain largely unknown.

To our knowledge, the data describing FGF13 expression in the adult human ovary is limited. Therefore, the objectives of our study were to detect the presence of follicular fluid (FF)-FGF13 and to evaluate the relationship between FF-FGF13 and oocyte developmental competence in patients undergoing in vitro fertilization/intracytoplasmic sperm injection (IVF/ICSI).

## Materials and methods

### Subjects and study design

A total of 75 patients aged 20–37 years undergoing first IVF/ICSI were recruited consecutively from Aug. 2014 to Aug. 2015 at the Center for Reproductive Medicine, Renji Hospital, School of Medicine, Shanghai Jiao Tong University. The patients had no medical history of hypertension, diabetes, hyperprolactinemia, thyroid disease, Cushing’s syndrome, or congenital adrenal hyperplasia. Patients who were using insulin-sensitizing drugs, oral contraceptives, corticosteroids, anti-androgens or gonadotropin-releasing hormone agonists/antagonists or who had undergone unilateral ovariectomy were excluded. Ultimately, 43 (57.3%) cases of PCOS and 32 (42.7%) cases of tubal infertility were included in the analyses.

The study protocol was approved by the Ethics Committee of Renji Hospital with informed consent.

### Collection of follicular fluid and biochemical measurements

Ovarian stimulation was performed using a GnRH antagonist protocol, and hCG (Lvzhu) was administered to trigger ovulation after adequate follicle development, as described previously [14]. Oocyte retrieval and follicular-fluid samples without blood contamination were collected under local anesthesia using vaginal ultrasound-guided punctures of follicles 36 h after hCG administration. Standard procedures were carried out for gamete-embryo handling, and embryo transfer was performed under abdominal ultrasonography guidance. The ICSI procedure was performed 4–6 h after oocyte retrieval.

Approximately 16–18 h after ICSI, the assessment of fertilization was performed. All embryos were scored as follows: Grade I, embryos with ≤5% fragmentation; Grade II, embryos with ≤20% fragmentation; Grade III, embryos with ≤50% fragmentation; and Grade IV, embryos with > 50% fragmentation [15]. On day 3, Grade I/II embryos were classified as high-quality embryos.

The levels of total testosterone (TT), estradiol (E2), progesterone (P4), luteinizing hormone (LH), follicle-stimulating hormone (FSH), and sex hormone-binding globulin (SHBG) in the follicular fluid were measured with chemiluminescence immunoassays (Elecsys autoanalyzer, Roche Diagnostics, Mannheim, Germany), and the free androgen index (FAI) was calculated with the following formula: FAI = 100 × TT/SHBG (nmol/L). The levels of interleukin-6 (IL-6), FGF13 and FGF21 in the follicular fluid were measured with enzyme-linked immunosorbent assay kits (CUSABIO Biotech Co., Ltd., Newark, DE, USA). The inter- and intra-assay coefficients of variation (CV) were < 10%.

### Diagnosis and definition

Polycystic ovaries were defined as follows: the presence of 12 or more follicles in each ovary measuring 2–9 mm in diameter and/or increased ovarian volume (> 10 mL for one or both ovaries) by transvaginal ultrasound. PCOS was defined when at least two of the following three criteria were met: oligo-ovulation or anovulation; clinical and/or biochemical signs of hyperandrogenism; and polycystic ovaries according to the revised Rotterdam consensus [16]. Thirty-two patients with tubal infertility who did not meet the diagnostic criteria for PCOS and had no family history of PCOS were defined as non-PCOS patients.

In the present study, “elevated” FF-FGF13 levels were defined as follicular levels in the upper quartile (i.e., ≥117.51 pg/mL). “Elevated” FF-TT levels were defined as follicular levels in the upper quartile (i.e., ≥51.90 nmol/L).

### Statistical analyses

Statistical analyses were performed with SAS version 9.2 (SAS Institute, Cary, NC, USA). Continuous variables due to skewed distributions are shown as medians (interquartile range). Categorical variables are shown as absolute numbers (percentages).

The baseline characteristics of the patients with and without elevated FF-FGF13 levels were described and compared using the Kruskal-Wallis tests for continuous variables and χ^2^ tests for categorical variables. Spearman correlations were performed to evaluate the relationships of FF-FGF13 to age, BMI, increased ovarian volume, FF-LH, FF-FSH, FF-TT, FF-FAI, FF-E2, FF-P4, FF-IL-6, and FF-FGF21 in the PCOS and non-PCOS patients. Spearman correlations and multiple linear regression analyses adjusted for age, BMI, FF-LH, and FF-FSH were performed to evaluate the associations between FF-FGF13 and oocyte developmental competence in the PCOS and non-PCOS patients.

Two-sided *P* values < 0.05 were considered statistically significant.

## Results

### General characteristics of the study patients

**Fig. 1:**
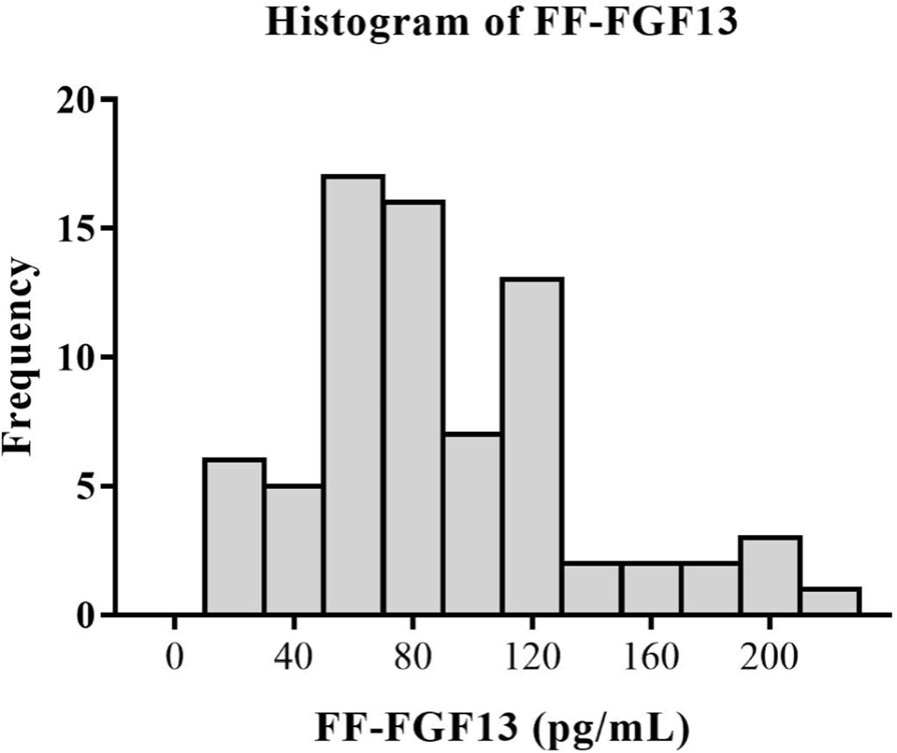
Distribution of FF-FGF13 levels among all patients in the study

A total 75 patients with a mean age of 27.7 ± 3.7 years were enrolled. The distribution of the FF-FGF13 levels was skewed with a median of 82.97 pg/mL (interquartile range 59.77–117.51 pg/mL). Figure 1 presents the histogram of the FF-FGF13 levels.

Table 1 presents the general characteristics of the patients with and without elevated FF-FGF13 levels. The patients with elevated FF-FGF13 levels had higher levels of FF-TT, FF-FAI, and FF-FGF21 than those without elevated FF-FGF13 levels (all *P* values < 0.05). The prevalence of increased ovarian volume was significantly higher among patients with elevated FF-FGF13 levels than among those without elevated FF-FGF13 levels (68.2% vs. 38.9%, *P* = 0.021). No significant differences were evident in terms of age, BMI, FF-LH, FF-FSH, FF-E2, FF-P4, or FF-IL6 between the patients with and without elevated FF-FGF13 levels.

**Table 1:** Comparison of the general characteristics of patients with and without elevated FF-FGF13

Characteristics	Elevated FF-FGF13		
	Yes	No	*P* values
FF-FGF13 (pg/mL)	143.22 (119.11–190.27)	70.03 (53.20–86.94)	─
n	19	56	─
Age (years)	28.0 (23.0–33.0)	27.0 (25.0–30.0)	0.69
BMI (kg/m^2^)	22.6 (20.2–24.0)	20.3 (19.0–23.5)	0.06
PCOS, n (%)	14 (73.7)	29 (51.8)	0.095
Increased ovarian volume, n (%)	13 (68.2)	21 (38.9)	0.021
FF-LH (IU/L)	2.73 (0.33–4.33)	0.94 (0.29–4.460)	0.57
FF-FSH (IU/L)	4.57 (3.50–6.78)	4.49 (3.36–5.55)	0.72
FF-TT (nmol/L)	51.90 (22.18–88.52)	24.71(16.32–43.44)	0.016
FF-FAI	1.86 (0.99–2.72)	0.95 (0.65–1.31)	0.014
FF-E2 (μg/L)	2028.00 (1218.00–3400.00)	1685.00 (1344.00–2353.00)	0.65
FF-P4 (mg/L)	27.62 (16.70–58.70)	39.88 (22.93–56.21)	0.21
FF-IL-6 (pg/mL)	5.39 (4.69–7.96)	5.74 (4.92–9.95)	0.71
FF-FGF21 (pg/mL)	16.48 (13.47–24.50)	13.97 (9.47–17.98)	0.023

Data are given as the median (interquartile range) for skewed variables or as the number (proportion) for categorical variables. “Elevated” FF-FGF13 levels were defined as follicular levels in the upper quartile (i.e., ≥117.51 pg/mL). P values were accessed using Kruskal-Wallis tests (continuous variables) and χ^2^ tests (categorical variables). *FGF13* fibroblast growth factor 13; *BMI* body mass index; *PCOS* polycystic ovary syndrome; *FF* follicular fluid; *LH* luteinizing hormone; *FSH* follicle-stimulating hormone; *TT* total testosterone; *FAI* free androgen index; E2: estradiol; P4: progesterone; IL6: interleukin-6; FGF21: fibroblast growth factor 21

**Fig. 2:**
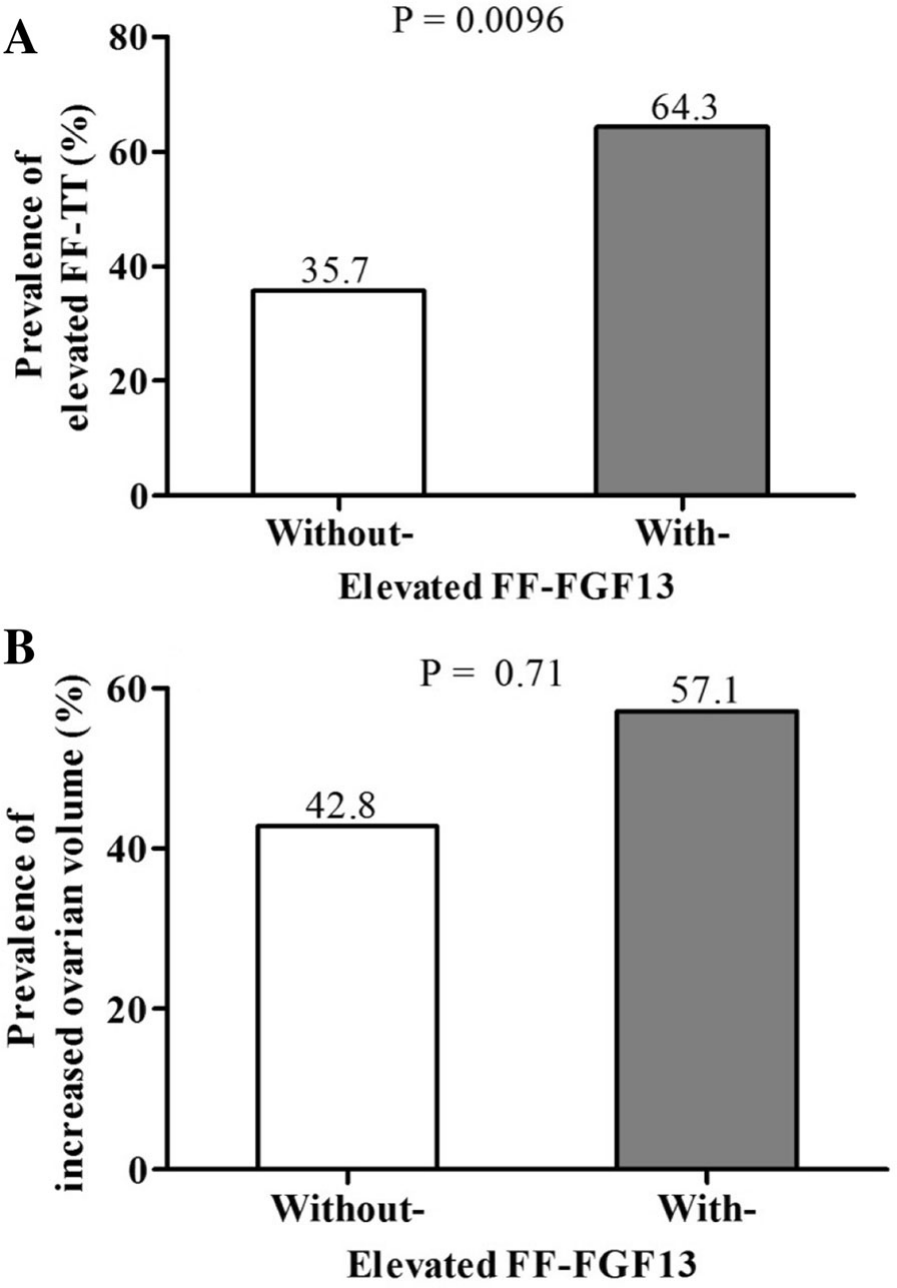
Prevalence of elevated FF-TT levels and increased ovarian volume in PCOS patients with and without elevated FF-FGF13 levels. Panel **a**: Prevalence of elevated FF-TT levels in PCOS patients with and without elevated FF-FGF13 levels. Panel **b**: Prevalence of increased ovarian volume in PCOS patients with and without elevated FF-FGF13 levels. *P* values were accessed using multiple logistic regression models adjusted for age, BMI, FF-FSH, and FF-LH

The prevalence of elevated FF-TT levels was significantly higher among PCOS patients with elevated FF-FGF13 levels than among those without elevated FF-FGF13 levels (64.3% vs. 35.7%, fully adjusted *P* = 0.0096). The prevalence of increased ovarian volume appeared to be higher among PCOS patients with elevated FF-FGF13 levels than among those without elevated FF-FGF13 levels (57.1% vs. 42.7%); however, the *P* value did not show a significant difference (Fig. 2).

### Factors associated with FF-FGF13 in patients with and without PCOS

As shown in Table 2, FF-TT and increased ovarian volume were positively correlated with FF-FGF13 in PCOS patients (*r* = 0.37, *P* = 0.013 and *r* = 0.33, *P* = 0.032). However, no significant relationships were evident between FF-FGF13 and FF-TT or increased ovarian volume among the non-PCOS patients (both *P* values > 0.05).

**Table 2:** Spearman correlations of risk factors associated with FF-FGF13 in PCOS and non-PCOS patients

	PCOS		Non-PCOS	
	r	*P* values	r	*P* values
Age (years)	−0.038	0.81	0.090	0.62
BMI (kg/m^2^)	0.10	0.50	0.37	0.041
Increased ovarian volume (YES = 1, NO = 0)	0.33	0.032	0.32	0.089
FF-LH (IU/L)	0.035	0.83	−0.0017	0.99
FF- FSH (IU/L)	−0.055	0.73	0.23	0.20
FF-TT (nmol/L)	0.37	0.013	0.035	0.85
FF-FAI	0.20	0.19	0.21	0.24
FF-E2 (μg/L)	0.14	0.3	−0.076	0.68
FF-P4 (mg/L)	−0.21	0.17	−0.20	0.27
FF-IL-6 (pg/mL)	−0.025	0.88	−0.036	0.85
FF-FGF21 (pg/mL)	0.32	0.035	0.33	0.063

r: correlation coefficient

### Associations between FF-FGF13 and oocyte developmental competence in patients with and without PCOS

As shown in Table 3, Spearman correlations revealed that FF-FGF13 was significantly correlated with the MII oocyte rate in the PCOS patients (*r* = − 0.42, *P* = 0.0055). The negative association persisted after the adjustments for age, BMI, FF-LH and FF-FSH in the multiple linear regression analysis (β = − 0.10, SE = 0.045 and *P* = 0.027). FF-FGF13 was significantly correlated with the fertilization rate in the non-PCOS patients (*r* = 0.52, *P* = 0.0037). However, the association disappeared in the multiple linear regression analysis (β = 0.078, SE = 0.099 and *P* = 0.44).

**Table 3:** Correlations between FF-FGF13 and oocyte developmental competence in PCOS and non-PCOS patients

	PCOS				Non-PCOS			
	r	*P* values	β ± SE	*P* values	r	*P* values	β ± SE	*P* values
NO. of oocytes retrieved	0.100	0.52	0.021 ± 0.17	0.90	−0.12	0.54	−0.060 ± 0.23	0.78
MII oocytes rate	−0.42	0.0055	−0.10 ± 0.045	0.027	0.096	0.61	−0.054 ± 0.084	0.53
Fertilization rate	−0.16	0.30	−0.060 ± 0.054	0.27	0.52	0.0037	0.078 ± 0.099	0.44
High-quality embryos rate	−0.043	0.78	0.014 ± 0.168	0.94	0.010	0.96	−0.15 ± 0.23	0.52

*P* values were obtained using Spearman correlations of risk factors associated with correlation and multivariable linear regression models adjusted for age, BMI, FF-FSH, and FF-LH. Data regarding oocyte developmental competence were missing for the non-PCOS group (*n* = 2)

r, correlation coefficient; β, regression coefficient

No associations were evident between FF-FGF21 and oocyte developmental competence for the PCOS patients when Spearman correlations were used (see Additional file 1).

## Discussion

This study is the first to report the presence of FGF13 in the follicular fluid of women undergoing IVF/ICSI. Moreover, FF-FGF13 was significantly associated with FF-TT and the MII oocyte rate in PCOS patients undergoing first IVF/ICSI in China.

Although FGF13 has been detected in the ovaries of rodents and cows, its expression in the ovaries of other species has remained unknown. FGF13 expression was first reported in the murine ovary by Helge Hartung [11]. Thereafter, FGF13 mRNA was observed in bovine theca and granulosa cells [17]. Afterward, the above results were confirmed by I.B. Costa et al. [13]. In the present study, FGF13 was detected in the follicular fluid of women undergoing first IVF/ICSI, which has direct implications for the roles of FGF13 in human ovarian function.

Despite similarities with other secreted FGFs, FGF13 has not been regarded as a secreted protein due to its lack of N-terminal signal sequences. However, our study demonstrated the presence of FGF13 in the follicular fluid of women undergoing IVF/ICSI. Moreover, the concentrations of FF-FGF13 were much higher than the levels of IL-6 in the follicular fluid, which is one of the endocrine factors. The results raise the possibility that FGF13 might be transported to the extracellular space. If so, the secretory mechanism of FGF13 might function in the same manner as that of FGF1 [18], despite the absence of a signal peptide. All of these possibilities require further investigation.

In the present study, FGF13 was associated with FF-TT in PCOS patients, raising the possibility of its essential role in the pathophysiological process of PCOS. Being different from other FGF family members, FGF13 cannot interact with FGF receptor tyrosine kinases [19]. The major downstream signaling pathways in the ovary function responsible for the association remain to be clarified. In previous research, FGF13 inhibited C2C12 cell differentiation by activating the extracellular signal-regulated kinases/mitogen-activated protein kinase (ERK/MAPK) pathway [20]. FGF13 was associated with the differential expression of the MAPK pathway in Sotos syndrome [21]. On the other hand, the MAPK pathway participated in the control of ovarian testosterone production [22]. Thus, we hypothesized that MAPK singling pathways were involved in the association between FGF13 and the testosterone secretion of the human ovary.

In addition to hyperandrogenism, the formation of ovarian interstitial fibrosis is a major cause of reproductive dysfunction in PCOS. In addition to MAPK singling pathways, the P38MAPK pathway, which is downstream of the FGF13 signaling pathway [23, 24], is involved in the expression of matrix metalloproteinase (MMP)2 and MMP9, which play important roles in extracellular matrix degradation in PCOS. Thus, FGF13 may be involved in ovarian interstitial fibrosis.

FGF21, a member of the family of secreted FGFs, is associated with insulin resistance [25, 26], an important aspect of the pathogenesis of PCOS. To explore its roles in PCOS, the levels of FGF21 in follicular fluid were examined in our study. The results showed that FGF21 was present in follicular fluid, but that it was not associated with oocyte developmental competence in PCOS patients undergoing IVF, implying that FGF21 in the follicular microenvironment might not be involved in oocyte developmental competence in PCOS patients.

The limitations of our study should be mentioned. First, the concentrations of FGF13 were examined in infertile patients after superovulation and not under normal physiological conditions. Second, the relatively small number of patients may have influenced the statistical power, and thus, a large-scale population study is needed in the future. Third, although possible covariates were included in the adjustments, some residual or undetected confounding factors could not be ruled out.

In summary, the present study reported the presence of FGF13 in the follicular fluid of women undergoing IVF/ICSI. Moreover, the relationships between FF-FGF13 and FF-TT, ovarian morphology and oocyte developmental competence imply that FF-FGF13 might be involved in the pathophysiological process of PCOS. FGF13 might be a promising intervention target in PCOS, as long as the potential mechanisms are clarified.

## Additional file

Additional file 1: Correlations between FF-FGF21 and oocyte development competence in PCOS patients. (DOCX 14 kb)
