# Supplementary material for: Intrafollicular fibroblast growth factor 13 in polycystic ovary syndrome: relationship with androgen levels and oocyte developmental competence
Source: J Ovarian Res. 2018 Sep 26;11:87. doi: 10.1186/s13048-018-0455-3 (PMC6158872; doi:10.1186/s13048-018-0455-3)
Supplement: Supplementary file 1 — Correlations between FF-FGF21 and oocyte development competence in PCOS patients. (DOCX 14 kb) [file 13048_2018_455_MOESM1_ESM.docx]

|  | r | P values |
| --- | --- | --- |
| NO. of oocytes retrieved | -0.064 | 0.69 |
| MII oocytes rate | -0.12 | 0.46 |
| Fertilization rate | 0.0020 | 0.99 |
| High-quality embryos rate | -0.041 | 0.79 |

**Additional file 1.** Correlations between FF-FGF21 and oocyte development competence in PCOS patients

P values were obtained using Spearman correlation. r, correlation coefficient.
